# Efficacy and Side Effects of Chinese Herbal Medicine for Menopausal Symptoms: A Critical Review

**DOI:** 10.1155/2012/568106

**Published:** 2013-01-13

**Authors:** Lian-Wei Xu, Man Jia, Roland Salchow, Michael Kentsch, Xue-Jun Cui, Hong-Yong Deng, Zhuo-Jun Sun, Lan Kluwe

**Affiliations:** ^1^Gynecology Department, Yueyang Integrated Traditional Chinese Medicine and Western Medicine Hospital of Shanghai University of Traditional Chinese Medicine, Shanghai 200437, China; ^2^HanseMerkur Traditional Chinese Medicine Centre, University Medical Centre Hamburg-Eppendorf, 20246 Hamburg, Germany; ^3^Department of Internal Medicine, University Teaching Hospital Itzehoe, 25524 Itzehoe, Germany; ^4^Clinical Evaluation Centre, Longhua Hospital of Shanghai University of Traditional Chinese Medicine, Shanghai 200032, China; ^5^Technology Information Centre, Shanghai University of Traditional Chinese Medicine, Shanghai 201203, China; ^6^Gynecology Department, Shuguang Hospital of Shanghai University of Traditional Chinese Medicine, Shanghai 200021, China; ^7^Laboratory for Research and Diagnostics, Departments of Maxillofacial Surgery and Neurology, University Medical Center Hamburg-Eppendorf, Martinistra**β**e 52, 20246 Hamburg, Germany

## Abstract

This study evaluates 23 (9 Chinese and 14 non-Chinese) randomized controlled trials for efficacy and side effects of Chinese herbal medicine on menopausal symptoms. Menopause was diagnosed according to western medicine criteria in all studies while seven Chinese studies and one non-Chinese study further stratified the participants using traditional Chinese medical diagnosis “Zheng differentiation.” Efficacy was reported by all 9 Chinese and 9/14 non-Chinese papers. Side effects and adverse events were generally mild and infrequent. Only ten severe adverse events were reported, two with possible association with the therapy. CHM did not increase the endometrial thickness, a common side effect of hormone therapy. None of the studies investigated long-term side effects. Critical analysis revealed that (1) high-quality studies on efficacy of Chinese herbal medicine for menopausal syndrome are rare and have the drawback of lacking traditional Chinese medicine diagnosis (Zheng-differentiation). (2) Chinese herbal medicine may be effective for at least some menopausal symptoms while side effects are likely less than hormone therapy. (3) All these findings need to be confirmed in further well-designed comprehensive studies meeting the standard of evidence-based medicine and including Zheng-differentiation of traditional Chinese medicine.

## 1. Introduction

Women can experience menopausal symptoms beginning in their mid-to-late forties [1]. It has been reported that almost 80% of women in western countries and more than 60% of Chinese women suffer from menopausal problems [2–4]. An American survey reports that approximately 25% of women require treatment [5]. Menopausal symptoms can last for 4-5 years or longer and can even be found in 9% of 72-year-old women [1, 3, 6, 7]. Menopausal syndrome not only has an impact on women's quality of life but also is associated with other health problems, for example, cardiovascular disease and osteoporosis in old age [8–10].

Clinical manifestations of menopausal syndrome have a multivariate feature, including vasomotor episodes, urogenital problems, sleep disturbance and mood disorders, uterine bleeding, somatic symptoms, vertigo and headaches, palpitations, skin formication, and sexual dysfunction [8, 11–16]. Among them, vasomotor symptoms, vaginal dryness, and sleep disturbance are most frequent and thus regarded as the most relevant problems, followed by mood symptoms and urinary complaints [3].

Hormone therapy is taken to be the most effective treatment for menopausal syndrome, but various disadvantages and side effects have been reported, including increased risk of breast and ovarian cancer, endometrial hyperplasia and carcinoma, stroke, and venous thromboembolism, especially for long-term therapy [17–25]. Furthermore, a significant proportion of menopausal women have contraindications to or are unwilling to use hormone therapy. Therefore, not only patients but also physicians are increasingly interested in complementary therapies using natural products with good effectiveness and fewer side effects [26, 27]. In USA, 82% physicians recommend herbal remedies to their menopausal patients [28].

Chinese herbal medicine (CHM), one of these natural product treatments with less side effect, has been widely used to disperse menopausal problems in China and other Asian countries [29–36]. However, in western society, the evidence of its efficacy is seen as unconvincing [3, 37–39]. Though there has been a large number of case reports and pilot clinical trials with various prescriptions in China in the past decades, they do not provide comparable, measurable, and reproducible evidence for efficacy of the treatments. On the other hand, randomized double-blind controlled trials in western medicine framework demand and favor homogeneity of participants, standardization of intervention, and quantitative measurements but lack consideration for Chinese medical Zheng features. 

**Figure 1:**
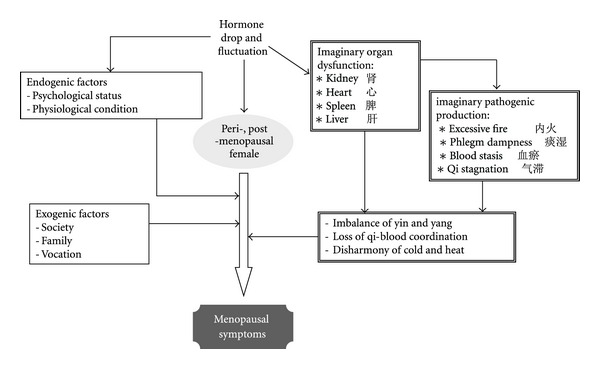
Illustration of the TCM understanding of menopausal symptoms.

Zheng differentiation (pattern differentiation, *辨证*), a syndrome stratification according to traditional Chinese medicine (TCM) diagnosis methods, plays the central role in the concept and practice of TCM. The logic of this TCM diagnosis differs fundamentally from that of the western scientific thinking. According to the TCM rationale, menopausal syndrome are caused by imaginary dysfunction of several organs such as kidney, liver, heart, and spleen, as well as imaginary pathogenic products induced by that dysfunction such as excessive fire, blood stasis, qi stagnation, and phlegm-dampness. The consequence is loss of coordination of qi and blood, disharmony of cold and heat, and imbalance of yin and yang (Figure 1) [40–44]. A Zheng differentiation of a menopausal case can be, for example, “aging-induced kidney dysfunction” or “kidney-based organ dysfunction” [40]. TCM therapies, both standardized and individualized, are adapted according to this kind of stratification. Efficacy of CHM on menopausal syndrome is thus also expected to rely on Zheng stratification [31, 45–47]. 

In this study, we evaluated more than 2000 published studies on efficacy of CHM for menopausal syndromes and critically analyzed 23 fit to our criteria, focusing on diagnosis, outcome measure, efficacy and side effects/adverse effects. We further discuss the role of Zheng-differentiation.

## 2. Materials and Methods

### 2.1. Databases and Search Strategy

Three Chinese electronic databases including VIP Database for Chinese Technical Periodicals (VIP), Chinese National Knowledge Infrastructure (CNKI), Chinese Biomedical Literature Database (CBM), and two major international electronic databases (Cochrane Library and MEDLINE) were searched. Specific search strategy for literatures was established for each of the five databases. The search strategy and terms for VIP, CNKI, and CBM were translated from Chinese. The search strategy for MDELINE was developed by modifying a published protocol of CHM for menopausal symptoms from Cochrane library [48]. Details of the search strategies and the abbreviation list are provided in the supplementary information available online at doi:10.1155/2012/568106. 

### 2.2. Inclusion and Exclusion Criteria

**Figure 2:**
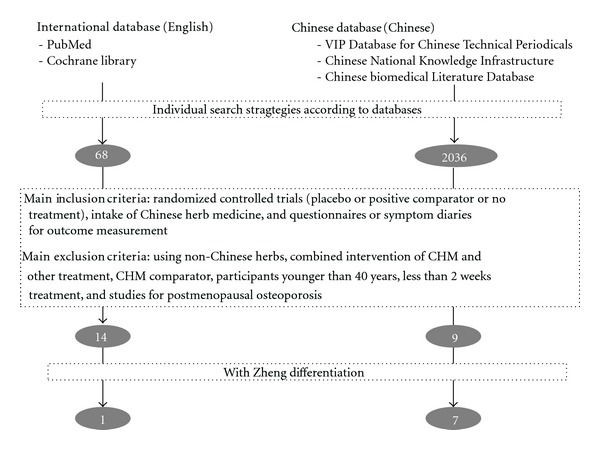
Number of studies on efficacy of CHM for menopausal syndrome at various stages of retrieval and selection process.

Randomized controlled trials of orally taken Chinese herbal medicine, including powders, liquid, pills, tablets, and capsules for treating physical or psychological menopausal symptoms published in Chinese or English were included. Kampo medicine (Japanese branch of traditional Chinese medicine), employing similar prescriptions of Chinese herbal medicine, was also considered [49–51]. Menopause included spontaneous ones and those induced by surgery, chemotherapy or radiotherapy. Control groups contained placebo, hormone therapy, other alternative medicine (e.g., SSRIs (selective serotonin reuptake inhibitors), oryzanol), acupuncture, and no treatment. Only studies with outcomes measured by quantitative questionnaires or participant's symptom diaries for menopausal symptoms were included (Figure 2).

Exclusion criteria were (1) using natural products such as soybean products, black cohosh (*Cimicifuga racemosa*), red clover (*Trifolium pratense*), St. John's wort (*Hypericum perforatum*), and other non-Chinese herbs, (2) combined interventions of Chinese herbal medicine with other treatments (hormone therapy, vitamins, minerals, cod-liver oil, evening primrose oil, acupuncture, acupoint, nutrition consultation, etc.), (3) using another CHM remedy as a comparator, (4) participants younger than forty, (5) interventions of less than two weeks, and (6) postmenopausal osteoporosis (Figure 2). 

### 2.3. Evaluation

Two independent specialists (Lian-Wei Xu and Man Jia) assessed the abstract and full-text literatures of all potential eligible trials meeting the inclusion criteria and summarized using data extraction forms from the selected studies. One of the two reviewers completed the forms which were subsequently confirmed by the others. Some of missing information was sought by contacting authors of the corresponding publications. The methodological quality of studies was evaluated using Jadad scale [52].

“Efficacy” for a CHM intervention is defined as (1) significant improvement compared to placebo or (2) similar improvement compared to standard therapy for either total scores or subscales of major relevant symptoms such as hot flushes and psychological parameters. 

## 3. Results 

### 3.1. Study Quality

A total of 2036 randomized controlled trials (RCTs) in Chinese databases and 68 in English databases were retrieved (Figure 2). Majority of studies were not blinded and many lacked adequate controls or comparators. Also lack of consideration for dropouts and lack of standardized outcome measures are frequent. The remaining total of 23 studies consisting of 9 Chinese and 14 non-Chinese met the inclusion and exclusion criteria and were further evaluated in following analysis [34, 53–74]. Jadad score varied from 1 to 4 (mean = 2.8) for the 9 Chinese papers and 2 to 5 (mean = 3.7) for the 14 non-Chinese papers (Table 2).

### 3.2. Diagnosis and Zheng Differentiation

**Figure 3:**
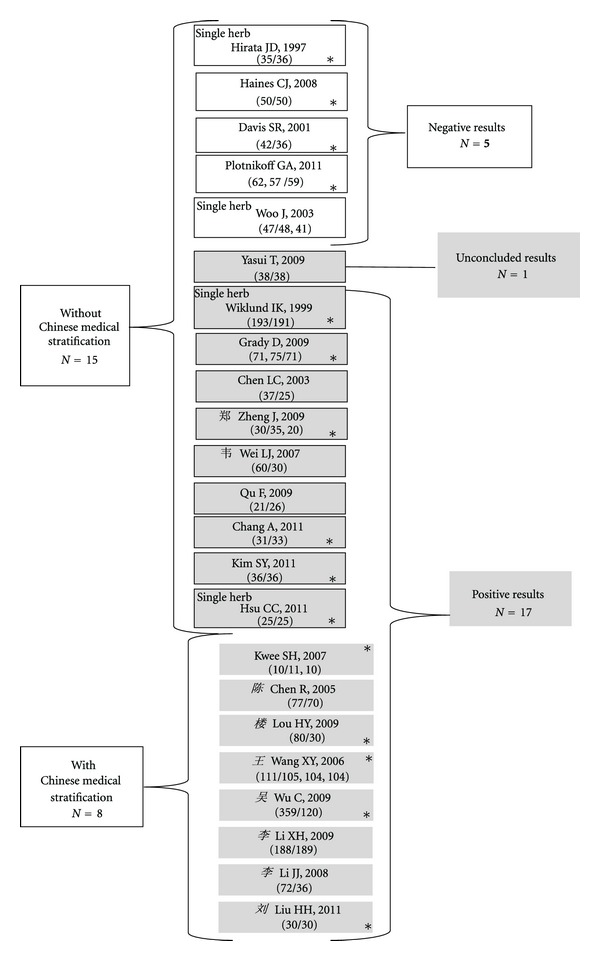
Classification of the 23 selected trials. The 4 single herb trials are marked at their upper-left corners, the 9 Chinese studies can be identified by the name of the first author in Chinese character. The 15 trials without Zheng differentiation were in boxes with single line and the 8 trials with Zheng differentiation were in italic. Boxes for trials with positive results are shaded. The fifteen trials applied placebo control marked with *. Others used positive comparators. Numbers in brackets are numbers of cases in CHM treatment/comparison groups.

In all these 23 RCTs, menopausal syndrome was diagnosed according to the standardized western medical criteria. Seven Chinese studies and one Netherlandish study further stratified the participants according to the TCM Zheng diagnosis (Figures 2 and 3, Table 1) [67–74]. Seven of studies considered yin deficiency and three specially mentioned kidney deficiency. These Zheng differentiation considered dysfunction of kidney, liver, and imbalanced pathogenic factors excessive liver qi, excessive fire, and blood stasis. The main Zhengs were (1) yin deficiency and excessive fire Zheng, (2) yin deficiency and excessive liver qi Zheng, (3) kidney (yin or yang) deficiency Zheng, and (4) spleen-kidney deficiency with blood stasis Zheng. Among these eight trials, one included all patients meeting western menopausal diagnosis and treated them individually according to the differential Zheng-differentiation [67]. The other seven included only patients meeting certain Zheng-differentiation for which the respective herbal mixture was formulated [68–74]. None of the studies described details of procedure of the Zheng-differentiation. 

### 3.3. CHM Interventions and Control

One study used hydrophilic concentration of individualized CHM prescribed according to the Zheng-differentiation of each participant [67]. All the other 22 used standard patented Chinese medicine of classical, modified classical or empirical prescriptions or single herb in granules, capsules, oral liquid, powder, or tablets (Table 1). Nineteen trials used mixed herbs while the other four used single herb (Figure 3, Table 1). 

The duration of the interventions was between eight weeks and two years (Table 1). One study had followup until four weeks after termination of the treatment [67]. 

Fifteen studies had placebo control, 9 used hormone therapy (Premelle, Premarin plus Medroxyprogesterone, Tibolone, or estradiol valerate), Paroxetine (SSRI), or vitamin E plus oryzanol as positive comparators (Table 1). 

### 3.4. Outcome Measure

All the 23 included trials used quantitative methodology to score and measure the extent of the menopausal symptoms and quality of life (Table 1). Kupperman Index and modified Kupperman Index are the most frequently used systematic measures (in 11/23 studies), especially in Chinese studies (8/9). Five Chinese trials employed the Chinese Medical Symptoms Scale corresponding to the TCM Zheng-differentiation. Other studies applied various scales including Greene Climacteric Scale, Menopause Rating Scale, Menopause Specific Quality of Life, Short-Form 36 Health Survey (SF-36), Pittsburgh Sleepiness Quality Scale, and Hamilton Depression Scale. Some of the trials provided scores of each symptom or domain separately while others gave the total scores for these standardized questionnaires. Six non-Chinese studies measured vasomotor symptoms by patient diary. 

### 3.5. Efficacy

All 9 Chinese and 8/14 non-Chinese studies reported positive effects of CHM while the other 6 non-Chinese studies did not find effectiveness. Positive effects included significant improvement (in total scores or in subscales of major relevant symptoms) compared to placebo and similar improvement compared to standard hormone therapy or other recognized alternative medicine. Reduction of hot flushes was the most frequently reported positive effect followed by improvement in total scores, benefits in depression, and other psychological measures. Generally, non-Chinese studies reported more details than Chinese ones.

Among the 9 Chinese studies, 5 employed placebo, 3 employed HRT, and one used Vit E plus oryzanol as positive comparators [61, 62, 68–74]. Majority of the Chinese studies reported only total scores of questionnaires but no data for subscales. Most studies declared that CHM improved scores of menopausal symptoms in comparison to placebo or reached similar effect of that of positive comparators (oryzanol or HRT). Only Wang et al. reported rather confusing results that CHM reduced total score of modified Kupperman index in the 8th week but not in the 12th week of the treatment [70].

One non-Chinese study observed significant improvement for Greene's scales for the CHM treatment group in comparison to baseline. However, most of these positive effects were significantly weaker than those of the Paroxetine treatment. The authors, thus, could not reach a conclusion for the efficacy of the CHM [57]. 

A total of 5 non-Chinese studies reported no efficacy (Figure 3). All these 5 studies employed placebo or no treatment as the comparators. Four studies reported substantial but similar improvements in both CHM and placebo groups [34, 53, 55, 56]. One did not find improvement at all in five major domains in CHM, HRT, and no treatment groups [54].

Eight out of the 9 Chinese studies and one non-Chinese study stratified patients according to their TCM-Zheng [67]. For example, Kwee et al. reported that individualized CHM for menopausal patients with Zheng-differentiation led to 29% reduction of average score of hot flushes compared to placebo [67]. In the study of 442 patients with yin deficiency and excessive liver qi, CHM mixture Jing Qian Ping granules significantly improved total scores of modified Kupperman Index and Chinese Medical Symptoms Scale compared to placebo (Table 1) [71]. 

Two of the 4 studies with single herb reported efficacy while the other two did not (Figure 3). 

A meta-analysis for efficacy of CHM was not feasible due to the variety of measurements of outcomes and the heterogeneity of the trials.

### 3.6. Safety and Adverse Effects

Eight trials systematically examined the endometrial thickness after the interventions and none of them found abnormal increase of thickness of endometrium by CHM. In contrast, increase of thickness of endometrium was reported in patients receiving hormone therapy which was used as a positive comparator in one study [62].

Nineteen trials monitored standard physiological functions and investigated adverse events or side effects of CHM (Table 1) [34, 53–61, 63–73]. Six trials (32%) reported no serious side effects or adverse events. Six of the remaining thirteen trials reported some adverse events which were, however, similar to those in corresponding placebo groups. Only one study reported more diarrhea in CHM group than in placebo (Table 1). The most common side effect was gastrointestinal symptoms including abdominal bloating or pain, epigastric discomfort, and stomach disorder in 8, followed by diarrhea in 7, headache in 4, nausea in 3, breast distension or pain in 2, abnormal vaginal bleeding in 2/19, and dizziness in 2 studies.

Only 10 severe adverse events were reported by three trials, among a total of 1837 participants (Table 2). One adverse event was per rectum bleeding, which may be possibly associated to the hot feature of Dang Gui Bu Xue Tang (DBT) [55]. Wiklund et al. reported 7 severe adverse events and stated that one of them was likely related to the CHM medication. However, no detailed information was available regarding feature of this event [58]. Two other serious adverse events were found in high dose of CHM of Grady et al.'s trial. One was idiopathic pancreatitis and the other one had occurred before the trial [59]. The paper did not mention the relationship between CHM intervention and idiopathic pancreatitis.

The longest trial over two years did not report serious adverse events [72]. None of the other studies investigated long-term side effect.

## 4. Discussion

### 4.1. Efficacy, Study Quality and Zheng Differentiation

To date, more than 2000 studies have been carried out concerning efficacy of CHM for menopausal syndrome, mostly in China and published in Chinese journals. However, only very few meet some of the standards of evidence-based medicine. We could only select 9 Chinese and 14 non-Chinese studies for evaluation. 

All Chinese studies reported effectiveness for CHM. However, these studies have generally low quality and lacked detailed data. In addition, the fact that Chinese journals traditionally publish only positive results seriously reduces reliability of the reported efficacies. 

Non-Chinese studies have generally better quality. However, most of these studies have the drawback of lacking consideration of Chinese medical features, especially Zheng-differentiation, the essential soul of TCM theory and practice. As in western medicine, CHM is also prescribed according to diagnosis which is based on a different way of interpretation and consideration of symptoms and endogenic/exogenic factors in a disordered and disharmonized menopausal female body (Figure 1). Thus, efficacy of CHM relies on Zheng-differentiation and may be less prominent in non-Chinese studies which do not apply Zheng-differentiation. Authors of a study carried out on American women indeed discussed that the lack of consideration of *sho *(similar to Zheng-differentiation) for participants may have contributed to the negative results [34]. 

Zheng-differentiation is a basic skill of TCM professionals who, however, often lack experience in randomized, blinded, and placebo-controlled clinical trials meeting the standard of evidence-based medicine in western countries. Cooperation of TCM and western medicine professionals is, thus, desirable for future studies on efficacy of CHM for menopausal in Chinese and non-Chinese females. Such studies will also help elucidating the role of Zheng-differentiation in TCM in general. 

### 4.2. Side Effect and Adverse Events

An important feature of CHM is the lack of increase of endometrial thickness, a common side effect of hormone therapy [62]. This can be well seen in several of the evaluated studies.

Other side effects of CHM are infrequent and generally mild. Among a total of 1837 treated cases, only ten severe adverse events were reported, though for eight of them there was no evidence of causal relation with the used CHM. Only two adverse events may have been related to the some components of the respective CHM: nausea in one case may be related to Ginseng [58] and per rectum bleeding in another case to the hot nature of Dang Gui Bu Xue Tang [55]. The most frequent side effects were mild gastrointestinal symptoms. 

The observation periods of the evaluated studies were generally short (around 12 weeks). Thus, long-term side effects known for CHM remain a central issue for future studies.

## 5. Conclusion 

Large number of studies have been carried out on efficacy of CHM for menopausal syndrome, but most of them lack adequate quality. CHM may be effective for at least some menopausal symptoms while its side effects are likely less than those of hormone therapy. However, all these findings need to be confirmed in further well-designed comprehensive studies which meet the standard of evidence-based medicine and include Zheng-differentiation of TCM. Cooperation of western medical and TCM professionals is essential. 

## Supplementary Material

Three Chinese databases, VIP, CNKI, CBM, and two English electronic databases, Cochrane Library, MEDLINE, were searched according to their special searching strategies. The details of the strategies, word strings and abbreviation are provided in the following supplementary information

## Figures and Tables

**Table:** (a)

Study	Number of case treatment/comparison	Age (year) treatment/comparison	Duration	Chinese herbal medicine, form and dose	Comparator	
					Placebo	Positive comparator
Hirata et al.,1997 [53]	35 (4)/36 (6)	52.2 ± 4.0/52.6 ± 6.0	6, 12, 24 weeks	Dong quai root, granular powder, 4.5 g/day	Yes	No

Woo et al.,2003 [54]	PL: 47 (5) HRT: 48 (2) No treatment: 41 (2)	PL: 57.4 ± 4.6 HRT: 56.2 ± 4.9 No treatment: 57.2 ± 4.8	3 months	*Pueraria lobata* (PL) powder, 100 mg/day	No	HRT: Premelle 1#/day

Haines et al.,2008 [55]	50 (5)/50 (11)	52.8 ± 4.9/51.2 ± 4.6	6 months	Dang Gui Bu Xue Tang (DBT) capsule 3 g/day	Yes	No

Davis et al.,2001 [56]	42 (14)/36 (9)	56.3 (54.3–58.3)/ 54.1 (52.6–55.5)	12 weeks	CHM granule	Yes	No

Plotnikoff et al., 2012 [34]	Low: 62 (11) High: 57 (13) Placebo: 59 (1)	Low dose: 53.7 ± 0.38 High dose: 53.6 ± 0.49 Placebo: 53.3 ± 0.38	12 weeks	TU-025 (keishibukuryogan) capsule, 7.5 g/day (low dose), 12.5 g/day (high dose)	Yes	No

Yasui et al.,2009 [57]	38 (3)/38 (6)	51.4 ± 5.1/50.5 ± 5.4	6 months	Kamishoyosan 7.5 g	No	Paroxetine 10 mg/day

Wiklund et al.,1999 [58]	193 (3)/191 (2)	53.3 ± 4.0/53.6 ± 4.0	16 weeks	Ginseng extract capsule 200 mg/day	Yes	No

Grady et al.,2009 [59]	Low: 71 (1) High: 75 (1) Placebo: 71 (2)	Low: 53.9 ± 2.6 High: 53.6 ± 3.0 Placebo: 53.7 ± 2.8	4, 8, 12 weeks	(1) MF101 powder, 5 g/day (2) MF101 powder, 10 g/day	Yes	No

Chen et al.,2003 [60]	37 (13)/25 (11)	50.17 ± 3.41/52.14 ± 5.33	16 weeks	Jia-Wey Shiau-Yau San (JWSYS), powder, 4 g, tid/day	No	Premelle 1^#^/day^▲▲^

*郑* Zheng et al.,2009 [61]	CHM: 30 (0) TCM package: 35 (0) Placebo: 20 (0)	CHM: 51.40 ± 2.57 TCM package: 51.17 ± 2.82 Placebo: 50.65 ± 2.32	4, 8, 12 weeks	Gengnianle Granule, 10 g, bid/day	Yes	TCM package^▲▲▲^

*韦* Wei and Luo,2007 [62]	60 (0)/30 (0)	40–55	3 months	Qianjinbayin 3 g bid/day	No	Premarin 0.625 mg/day + Medroxyprogesterone 6 mg/day (15th–28th day)

Qu et al.,2009 [63]	21 (0)/26 (0)	48.7 ± 8.1/50.4 ± 9.3	12 weeks	Gengnianle decoction 200 mL/day	No	Tibolone 2.5 mg/day

Chang et al., 2012 [64]	31 (2)/33 (1)	53.2 ± 5.7/54.1 ± 5.9	6, 12 weeks	EstroG-100, 1^#^ bid/day	Yes	No

Kim et al., 2012 [65]	36 (5)/36 (4)	52.98 ± 3.04/55.01 ± 3.67	12 weeks	Red ginseng capsule 1 g tid/day	Yes	No

Hsu et al.,2011 [66]	25 (0)/25 (0)	51.92 ± 2.97/53.08 ± 3.00	6, 12 months	Dioscorea extracts 12 mg bid/day	Yes	No

Kwee et al.,2007 [67]	CHM: 10 (1) HRT: 11 (0) Placebo: 10 (0)	CHM: 53.2 (51.5–55.0) HRT: 53.6 (50.0–57.3) Placebo: 54.9 (51.6–58.2)	12 weeks, plus 4-week followup	(i) Modified Zhi Bai Di Huang Wan (ii) Individualized hydrophilic concentration	Yes	Premelle 1^#^/day

*陈* Chen et al.,2005 [68]	77 (12)/70 (12)	50.79 ± 4.45/50.43 ± 4.16	1, 3 months	Kuntai capsule 2 g tid/day	No	E_2_V 0.5 mg/day^▲▲▲▲^

*楼* Lou et al.,2009 [69]	80 (11)/30 (3)	52.69 ± 3.12/51.85 ± 2.92	8 weeks	Xian ling gu bao capsule 3^#^ bid/day	Yes	No

*王* Wang et al.,2006 [70]	CHM: 111 (—) CHM + qing zhi: 105 (—) Qing zhi: 104 (—) Placebo: 104 (—)	No detail^▲^	4, 8, 12 weeks	(1) Geng nian ning C:C tid/day for kidney yin deficiency (2) Bushen oral liquid, 1 bottle bid/day for kidney yang deficiency	Yes	(1) CHM + Chinese medical qing zhi therapy^▲▲▲▲▲^ (2) Chinese medical qing zhi therapy

*吴* Wu et al.,2009 [71]	359 (23)/120 (14)	45–55	8 weeks	Jing qian ping granule 4 g tid/day	Yes	No

*李* Li et al.,2009 [72]	188 (53)/189 (96)	48.8 ± 2.93/48.6 ± 2.74	2 years	Bushen Zhuanggu granule 100 g bid/day × 6 months, then 100 g/day	No	Premarin 0.625 mg/day + Medroxyprogesterone 2 mg/day

*李* Li et al.,2008 [73]	72 (0)/36 (0)	49.25 ± 2. 38/49.15 ± 2.68	3 months	Gengnianningshentang decoction	No	Vit E 100 mg bid + oryzanol 20 mg tid

*刘* Liu et al.,2011 [74]	30 (5)/30 (4)	50.63 ± 3.8/50.63 ± 4.65	12 weeks	Ziyin Jianghuo Fang (ZYJHF) granules	Yes	No

Total amount	1837/1609	//	/	/	/	/

—: did not report.

^▲^Including patients with age from 45 to 55, according to the defined inclusion criteria published by Chinese Ministry of Health, 1997 [75].

^▲▲^Premelle: 1 tablet includes 0.625 mg conjugated oestrogen, 5 mg medroxyprogesterone.

^▲▲▲^TCM package: CHM + Chinese medical psychological therapy + Taiji.

^▲▲▲▲^E_2_V: estradiol valerate.

^▲▲▲▲▲^Chinese medical qing zhi therapy: Chinese medical psychological therapy, *情志疗法*.

/: blanket.

**Table:** (b)

Study	Outcome measure	Outcomes			Efficacy
		Treatment versus placebo/no treatment	Treatment versus positive comparator	Treatment versus baseline
Hirata et al.,1997 [53]	(a) Kupperman Index (b) Diary of number of vasomotor symptoms	No significant improvement for (a) and (b)	/	About 25%–30% reduction for (a) and (b), score of (a) from 19.0 ± 8.4 to 12.2 ± 5.2 (*P* < 0.001), number of vasomotor episodes per week from 47.3 ± 39.9 to 30.7 ± 21.7 (*P* > 0.05)	No

Woo et al.,2003 [54]	(a) Menopausal symptoms questionnaire (b) Short Form 36 Health Survey (c) Mini-Mental State Examination (MMSE)	*No improvement for majority items of (a) and (b); *more improvement for cognitive function	Similar change for (a), (b), and (c)	*No improvement for five domains of (a); *for (b), percentage change 10.0 ± 20.5 for physical functioning, 28.6 ± 67.5 for role physical, 16.5 ± 48.0 for bodily pain, 13.5 ± 69.7 for general health, 25.7 ± 52.8 for vitality, 13.0 ± 58.9 for social functioning, 0.5 ± 82.3 for role emotional, and 13.2 ± 23.0 for mental health; *percentage increase 3.4 ± 8.5 for (c)	No

Haines et al.,2008 [55]	(a) Self-reported daily diary for vasomotor symptoms (b) Menopause-specific quality of life	*No significant difference for mild, moderate, and severe hot flushes as well as night sweats of (a); *similar improvement for four domains of (b)	/	*Improvement for number of mild hot flushes from 18.9 ± 23.5 to 8.6 ± 17.1 per month (*P* = 0.002); *improvement for sexual domain of (b) from 3.49 ± 1.96 to 2.73 ± 1.80 (*P* < 0.01)	No

Davis et al.,2001 [56]	(a) Diary of the frequency of vasomotor symptoms (b) Menopause-specific quality of life	*The frequency of vasomotor symptoms reduced but with similar improvement; *similar reduction for scores of four domains of (b)	/	*More than 40% reduction in the frequency of vasomotor symptoms (*P* = 0.001); *improvement for physical, vasomotor, and sexual domains of (b)	No

Plotnikoff et al., 2012 [34]	(a) Daily Mayo Hot Flash Symptom Diary; (b) Greene Climacteric Scale; (c) Pittsburgh Sleepiness Quality Scale	Similar improvement for (a), (b), and (c) without significant difference (*P* > 0.05)	/	*40% Improvement for (a) in low-dosage group, 38% in high-dosage group (*P* < 0.001); *significant reduction for the mean scores of (b) (*P* < 0.001); *improvement for (c) and its subscales (*P* < 0.001), except sleep medication use	No

Yasui et al.,2009 [57]	Greene Climacteric Scale	/	Less improvement for psychological (*P* = 0.0007), vasomotor(*P* = 0.05)and total score (*P* = 0.0002), no difference for somatic subscore (*P* = 0.167)	Improvement for psychological, somatic, and vasomotor subscores and total score (*P* < 0.0001)	Unconcluded

Wiklund et al.,1999 [58]	(a) Psychological general well-being Index; (b) women's health questionnaire; (c) visual analogue scales	*Slightly better overall symptomatic relief (*P* < 0.1); *significant better improvement in depression and well-being subscales (*P* < 0.05); *no significant effects for (b) and (c) or the physiological parameters, including vasomotor symptoms	/	*Improvement for total score of (a) and anxiety, depression, well-being, self-control, health, vitality subscores; *improvement for vasomotor and somatic symptoms, sleep and menstrual problems, depression, anxiety, attraction, cognitive function scores and total score of (b); *improvement for total score and vasomotor, emotional symptoms of (c), reduction of vasomotor from 48.8 ± 22.2 to 34.3 ± 26.3 (*P* = 0.0001)	Yes

Grady et al.,2009 [59]	(a) Diary of the frequency and severity of vasomotor symptoms; (b) Short Form 36 Health Survey; (c) Female Sexual Function Index	For high-dose group, 33% greater improvement for frequency of mild hot flush (*P* = 0.02); 67% reduction in the number of awake-sleep by hot flushes per week (*P* = 0.05); 16.2% more improvement for 50% reduction of hot flushes (*P* = 0.03)	*/ *	In high-dosage group, 48% reduction for number of hot flushes per week, 67% reduction for number of awake sleep, and 47% for 50% reduction of frequency of hot flushes, respectively 37%, 58%, and 39% in low-dosage group, 37%, 44%, and 31% in placebo group	Yes

Chen et al.,2003 [60]	Greene Climacteric Scale	/	*Similar improvement for psychological (anxiety and depression), somatic and vasomotor subscores as well as total score (*P* > 0.05); *less improvement for sexual dysfunction (*P* < 0.05)	Improvement for psychological, anxiety, depression, somatic and vasomotor subscores and total score (*P* < 0.05)	Yes

*郑* Zheng et al.,2009 [61]	(a) Modified Kupperman Index; (b) Chinese Medical Symptoms Scale	Improvement for total scores of two scales (*P* < 0.05)	less reduction than TCM package for total scores of (a) and (b) (*P* < 0.05)	Improvement for (a) and (b) at weeks 8 and 12 (*P* < 0.05)	Yes

*韦* Wei and Luo,2007 [62]	Modified Kupperman Index	/	Similar for total score (*P* > 0.05)	Improvement for total score from 30.46 ± 6.84 to 8.26 ± 9.22 (*P* < 0.05)	Yes

Qu et al.,2009 [63]	Hamilton Depression Scale	/	*No significant difference for total score (*P* > 0.05); *inimitably improved insomnia middle and anxiety (somatic), no improvement for work and activities, agitation	Improvement for depressed mood, feeling of guilt, suicide, insomnia early, insomnia middle, anxiety (psychological and somatic) subscores (*P* < 0.05)	Yes

Chang et al., 2012 [64]	Kupperman menopause Index	Magnificently improved vasomotor, numbness and tingling, insomnia, nervousness, feeling blue and depressed, dizzy spells, tired feelings, rheumatic pain, sensation of crawling on the skin, vaginal dryness (*P* < 0.01), no improvement for headache and palpitation (*P* > 0.05)	/	Improvement for all subscales at week 12 (*P* < 0.05)	Yes

Kim et al., 2012 [65]	(a) Kupperman Index; (b) Menopause Rating Scale	*Better for hot flash subscore (*P* = 0.046) and total score of (a) (*P* = 0.032); *significant reduction for total score (*P* = 0.035) but no improvement for hot flash of (b) (*P* = 0.121)	/	*Improvement for total score of (a) from 18.93 ± 11.28 to 13.32 ± 10.15 (*P* = 0.021), hot flash from 5.25 ± 3.59 to 3.51 ± 2.36 (*P* = 0.032); *improvement for total score of (b) from 12.45 ± 8.79 to 8.32 ± 6.75 (*P* = 0.027), hot flash from 1.85 ± 1.15 to 1.10 ± 0.79 (*P* = 0.096)	Yes

Hsu et al.,2011 [66]	Greene Climacteric Scale	More improvement for feeling tense or nervous (*P* = 0.07), insomnia (*P* = 0.004), excitable (*P* = 0.047), musculoskeletal pain (*P* = 0.019) after 12 months	/	More than 90% improvement in almost all parameters (except sexual function) at months 6 and 12	Yes

Kwee et al.;2007 [67]	(a) Diary for frequency of vasomotor symptoms; (b) Short Form 36 Health Survey	Improvement for hot flushes with 29% greater average score, more efficacy for weeks 5, 7–11 (*P* < 0.05)	*Less improvement for hot flushes with 50% average score, especially at weeks 4–13 (*P* < 0.01); *no improvement for hot flush reduction of (b)	Improvement for score of (a) at weeks 5, 7–11, no improvement for (a) and (b) at week 16	Yes

*陈* Chen et al.,2005 [68]	Modified Kupperman Index	*/ *	Similar improvement for hot flush score and total score (*P* > 0.05)	Improvement for total score from 25.05 ± 8.01 to 8.73 ± 6.06	Yes

*楼* Lou et al.,2009 [69]	(a) Chinese Medical Symptoms Scale	More reduction for total scores of (a) (*P* < 0.01)	*/ *	Improvement for total score of (a) from 15.58 ± 3.45 to 5.66 ± 1.24 (*P* < 0.01)	Yes

*王* Wang et al.,2006 [70]	(a) Modified Kupperman Index; (b) Chinese Medical Symptoms Scale	*More improvement for total score of (a) at 8th week (*P* < 0.05) but not better at 12th week (*P* > 0.05); *better for total score of (b) at 8th and 12th weeks (*P* < 0.05)	*More improvement for total score of (a) and (b) (*P* < 0.05)	—	Yes

*吴* Wu et al.,2009 [71]	(a) Chinese Medical Symptoms Scale; (b) modified Kupperman Index	More improvement for total scores of (a) and (b) at week 8 (*P* < 0.05)	*/ *	—	Yes

*李* Li et al.,2009 [72]	Kupperman Index	*/ *	Better for total score (*P* < 0.01)	Improvement for total score from 26.67 ± 5.02 to 20.36 ± 4.03 (*P* < 0.01)	Yes

*李* Li et al.,2008 [73]	Modified Kupperman Index	*/ *	More reduction for total score (*P* < 0.01)	Improved total score from 26.05 ± 3.31 to 7.75 ± 2.851 (*P* < 0.05)	Yes

*刘* Liu et al.,2011 [74]	(a) Number of hot flushes and sweat; (b) Chinese Medical Symptoms Scale; (c) Kupperman Index	*More improvement for (a) (*P* < 0.05), (b) (*P* < 0.01), and (c) (*P* < 0.01)	*/ *	*Reduction for number of hot flushes per day from 7.10 ± 2.06 to 2.20 ± 1.79 (*P* < 0.05), number of sweats per day from 7.07 ± 1.87 to 2.13 ± 1.68 (*P* < 0.05); *improvement for total score of (b) from 26.67 ± 5.49 to 8.50 ± 3.51 (*P* < 0.01); *improvement for total score of (c) from 25.47 ± 5.45 to 6.80 ± 2.61 (*P* < 0.01)	Yes

**Table:** (c)

Study	Adverse events/side effects	Thickness of endometrium	Zheng differentiation	Prescriptions
Hirata et al.,1997 [53]	Burping, gas, headache (similar to placebo)	No increase at 24 weeks, no difference to placebo	No	Dang gui (*Angelica sinensis*) root

Woo et al.,2003 [54]	Urticaria	—	No	Ge gen (*Pueraria lobata*)

Haines et al.,2008 [55]	Constipation, epigastric discomfort, hypercholesterolemia, per rectum bleeding (SAE) (no difference to placebo)	—	No	Dang gui (*Angelicae sinensis*) : huang qi (*Astragalus membranaceus*) = 1 : 5

Davis et al.,2001 [56]	Abdominal bloating, lower abdominal pain and loose stools, headache, joint pain, dizziness (no difference to placebo)	—	No	Shu di huang (*Rehmannia glutinosa*) 15 g, shan zhu yu (*Cornus officinalis*) 10 g, shan yao (*Dioscorea opposita*) 12 g, ze xie (*Alisma orientalis*) 8 g, dan pi (*Paeonia suffruticosa*) 8 g, fu shen (*Poria cocos*) 12 g, chen pi (*Citrus reticulata*) 5 g, di gu pi (*Lycium chinensis*) 20 g, he huan pi (*Albizia julibrissin*) 15 g, suan zao ren (*Zizyphus jujuba*) 10 g, han lian cao (*Eclipta prostrata*) 15 g, and nu zhen zi (*Ligustrum lucidum*) 10 g

Plotnikoff et al., 2012 [34]	Prevalent diarrhea (more than in placebo)	—	No	Rou gui (*Cinnamomum cassia Blume*), bai shao (*Paeonia lactiflora* Palls), tao ren (*Prunus persica *Batsch), fu ling (*Poria cocos Wolf*), and dan pi (*Paeonia suffruticosa *Andrews)

Yasui et al.,2009 [57]	Diarrhea	—	No	*Angelica root*, *Atractylodis lanceae rhizome*, *Peony root*, *Bupleurum root*, *Hoelen*, *Glycyrrhiza root*, *Moutan bark*, *Gardenia fruit*, *Ginger rhizome,* and *Mentha herb *

Wiklund et al.,1999 [58]	Headache/migraine, diarrhea/gastrointestinal system disorders, nausea, seven SAE (no difference to placebo)	No increase	No	Standardized extracts of ren shen (*Ginseng root*)

Grady et al.,2009 [59]	Loose stools, vaginal bleeding, idiopathic pancreatitis (SAE) (no difference to placebo)	No difference among three groups	No	Ban zhi lian (*Herba Scutellaria barbata*) 30 g, shan dou gen (*Radix Sophora subprostratae*) 15 g, zhi mu (*Radix Anemarrhenae*) 12 g, hei dou (*Semen Glycine sojae*) 20 g, gan cao (*Radix Glycyrrhiza*) 8 g, da huang (*Rhizoma Rhei*) 8 g, fu xiao mai (*Fructus Tritici levis*) 15 g, huang qi (*Radix Astragali*) 12 g, sheng di huang (*Radix Rehmannia*) 12 g, nu zhen zi (*Fructus Ligustri lucidi*) 15 g, suan zao ren (*Semen Zyziphi spinozae*) 10 g, lian zi xin (*Plumula Nelumbinis*) 10 g, fu ling (*Poria Cocos*) 10 g, ze xie (*Rhizoma Alismatis*) 10 g, mu dan pi (*Cortex Moutan radicis*) 8 g, shan zhu yu (*Fructus Corni*) 10 g, huai niu xi (*Radix Achyranthis*) 10 g, mu li (*Concha Ostrea*) 12 g, tian men dong (*Radix Asparagi*) 12 g, ge gen (*Radix Pueraria*) 10 g, bai zhu (*Radix Atractylodis macrocephala*) 10 g, and yin yang huo (*Herba Epimedii*) 8 g

Chen et al.,2003 [60]	Bloated abdomen, unusual vaginal bleeding, nausea and cough (no mention for the difference)	—	No	Dang gui (*Angelicae Radix*) 4 g, bai zhu (*Atractylodis Rhizoma*) 4 g, bai shao (*Paeoniae Radix*) 4 g, chai hu (*Bupleuri Radix*) 4 g, fu ling (*Poria cocos Wolf*) 4 g, gan cao (*Glycyrrhizae Radix*) 2 g, mu dan pi (*Moutan Bark*) 2.5 g, zhi zi (*Gardeniae Fructus*) 2.5 g, gan jiang (*Zingiberis Rhizoma*) 4 g, and bo he (*Menthae Herba*) 2 g

*郑* Zheng et al.,2009 [61]	Diarrhea (one case)	—	No	Huang lian (*Rhizoma Coptidis*), mai dong (*Radix Ophiopogonis*), mu dan pi (*Moutan Bark*), and other herbs

*韦* Wei and Luo,2007 [62]	—	(i) No increase in treatment group (ii) Increase in hormone therapy group	No	Qian jin ba (*Moghania philippinensis*), ge jei (*Gekko gecko*), mei gui hua (*Rosa rugosa*), nuo dao gen (*Radix Oryzae Glutinosae*), and other herbs

Qu et al.,2009 [63]	No serious side effect	—	No	Zhen zhu mu (*C. Margaritifera*) 15 g, suan zao ren (*Semen Z. Spinosae*) 8 g, bai zi ren (*Semen Platycladi*) 12 g, yuan zhi (*of radix polygalae*) 10 g, he huan pi (*cortex albiziae*) 8 g, huang qi (*radix astragali*) 15 g, xi yang shen (*radix codonopsis*) 10 g, shan yao (*radix dioscoreae*) 15 g, tu si zi (*semen cuscutae*) 15 g, and nv zhen zi (*fructus L. Lucidi*) 10 g

Chang et al., 2012 [64]	No adverse events	—	No	*Cynanchum wilfordii, Phlomis umbrosa,* and *Angelica gigas *

Kim et al., 2012 [65]	—	—	No	Hong shen (*red ginseng*)

Hsu et al.,2011 [66]	No serious adverse events except soft stools, nausea (mild and transient) (no mention for the difference)	No increase	No	Shan yao (*Dioscorea alata*)

Kwee et al.,2007 [67]	No serious adverse events	—	Kidney yin deficiency	Zhi mu (*Rhizoma Anemarrhenae*) 5.1%, huang bai (*Cortex Phellodendri*) 5.1%, shu di huang (*Radix Rehmanniae praeparata*) 20.5%, shan zhu yu (*Fructus Corni*) 10.3%, shan yao (*Rhizoma Dioscoreae oppositae*) 10.3%, fu ling (*Sclerotium Poriae albae*) 7.7%, duan long gu (*Os Draconis ustum*) 10.3%, duan mu li (*Concha Ostreae usta*) 10.3%, mu dan pi (*Cortex Moutan radicis*) 7.7%, and ze xie (*Rhizoma Alismatis*) 7.7%, and gou qi zi (*Fructus Lycii*) 5%—without details for the modification

*陈* Chen et al.,2005 [68]	Gastrointestinal symptoms, breast distension and pain, and vaginal bleeding (no mention for the difference)	No increase	Yin deficiency with excessive fire	Shu di huang (*Radix Rehmanniae praeparata*), huang lian (*Rhizoma Coptidis*), bai shao (*Radix Paeoniae*), e jiao (*Colla Corii asini*), huang qin (*Radix Scutellariae*), and fu ling (*Poria*)

*楼* Lou et al., 2009 [69]	No serious side effect	No increase	Yin deficiency with excessive fire	Yin yang huo (*Herba Epimedii*), other herbs (without details)

*王* Wang et al.,2006 [70]	No serious side effect	No increase	Kidney yin deficiency/kidney yang deficiency	Geng nian ning capsule: shu di huang (*Radix Rehmanniae praeparata*), fu ling (*Poria*), huang lian (*Rhizoma Coptidis*), e jiao (*Colla Corii asini*), other herbs bu shen oral liquid: shu di huang (*Radix Rehmanniae praeparata*), nu zhen zi (*Fructus Ligustri lucidi*), yin yang huo (*Herba Epimedi*), and other herbs (without details)

*吴* Wu et al.,2009 [71]	Stomach disorder, breast distension (no difference to placebo)	—	Yin deficiency with excessive liver qi	Bai shao (*Radix Paeoniae*), xiang fu (*Rhizoma Cyperi*), chuan lian zi (*Fructus Toosendan*), chai hu (*Radix Bupleuri*), and other six herbs

*李* Li et al.,2009 [72]	No serious adverse events	—	Spleen-kidney deficiency with blood stasis	Shan zhu yu (*Cornus officinalis*), lu jiao jiao (*Colla Cornus Cervi*), gui ban jiao (*Chinemys reevesii*), rou gui (*Cinnamomum cassia Blume*), ba ji tian (*Radix Morindae officinalis*), yin yang huo (*Herba Epimedi*), bai shao (*Radix Paeoniae*), san leng (*Rhizoma Sparganii Stoloniferi*), ze xie (*Rhizoma Alismatis*), shui zhi (*Hirude nipponica *Whitman), yu jin (*Curcuma aromatica Salisb*), gu sui bu (*Rhizoma Drynariae*), shan yao (*Dioscorea opposita*), and other herbs (without details)

*李* Li et al.,2008 [73]	Headache, dizziness, epigastric discomfort (no difference to vitamin E plus oryzanol treatment)	—	Yin deficiency with excessive fire	Sheng di huang (*Radix Rehmannia*) 30 g, shan zhu yu (*Cornus officinalis*) 15 g, nu zhen zi (*fructus L. Lucidi*) 15 g, han lian cao (*Eclipta prostrata*) 15 g, gou qi zi (*Fructus Lycii*) 15 g, tu si zi (*semen cuscutae*) 15 g, dan shen (*Salvia miltiorrhiza*) 15 g, di gu pi (*Lycium chinensis*) 15 g, gui ban (*Carapax Testudinis*) 15 g, zhen zhu mu (*C. Margaritifera*) 15 g, wu wei zi (*Schisandra chinensis*) 10 g, yuan zhi (*Radix Polygalae*) 10 g, and yin yang huo (*Herba Epimedi*)10 g

*刘* Liu et al.,2011 [74]	—	—	Yin deficiency with excessive fire	Gou teng (*Gambir Plant*)15 g, lian zi xin (*Plumula Nelumbinis*) 5 g, huang lian (*Rhizoma Coptidis*) 3 g, suan zao ren (*Semen Z. Spinosae *) 15 g, fu xiao mai (*Fructus Tritici levis*) 30 g, dan shen (*Salvia Miltiorrhiza*) 10 g, sheng di huang (*Radix Rehmannia*) 10 g, and shan zhu yu (*Fructus Corni*) 9 g

**Table 2:** Quality of the included studies.

Source (author, year)	English/Chinese	Jadad scale score			
		Randomization	Double blinding	Withdrawals/dropouts	Total score
Hirata et al.,1997 [53]	English	2	1	1	4
Woo et al.,2003 [54]	English	1	1	1	3
Haines et al.,2008 [55]	English	2	2	1	5
Davis et al.,2001 [56]	English	2	2	1	5
Plotnikoff et al., 2012 [34]	English	2	1	1	4
Yasui et al., 2009 [57]	English	1	0	1	2
Wiklund et al.,1999 [58]	English	1	2	1	4
Grady et al.,2009 [59]	English	2	2	1	5
Chen et al.,2003 [60]	English	1	0	1	2
Qu et al.,2009 [63]	English	2	0	0	2
Chang et al., 2012 [64]	English	2	1	1	4
Kim et al., 2012 [65]	English	2	1	1	4
Hsu et al.,2011 [66]	English	1	2	0	3
Kwee et al.,2007 [67]	English	2	2	1	5

Mean score	/	/	/	/	3.7

*郑* Zheng et al.,2009 [61]	Chinese	2	0	0	2
*韦* Wei and Luo,2007 [62]	Chinese	2	0	0	2
*陈* Chen et al.,2005 [68]	Chinese	1	1	1	3
*楼* Lou et al.,2009 [69]	Chinese	2	2	0	4
*王* Wang et al.,2006 [70]	Chinese	2	2	0	4
*吴* Wu et al.,2009 [71]	Chinese	2	2	0	4
*李* Li et al.,2009 [72]	Chinese	2	0	1	3
*李* Li et al.,2008 [73]	Chinese	2	0	0	2
*刘* Liu et al.,2011 [74]	Chinese	1	0	0	1

Mean score	/	/	/	/	2.8
Total mean score	/	/	/	/	3.3
