# Supplementary material for: Efficacy and Side Effects of Chinese Herbal Medicine for Menopausal Symptoms: A Critical Review
Source: Evid Based Complement Alternat Med. 2013 Jan 13;2012:568106. doi: 10.1155/2012/568106 (PMC3551256; doi:10.1155/2012/568106)
Supplement: Supplementary file 1 — Three Chinese databases, VIP, CNKI, CBM, and two English electronic databases, Cochrane Library, MEDLINE, were searched according to their special searching strategies. The details of the strategies, word strings and abbreviation are provided in the following supplementary information [file 568106.f1.docx]

Supplementary information

1. search strategies for literature databases:

(1) VIP (from 1979 to March 2012) (the strategies are translated from Chinese)

Any field: menopausal syndrome AND (Chinese medicine OR Chinese herb OR Chinese herbal medicine) AND clinical AND randomized

Title/key word/ abstract: menopausal syndrome AND (Chinese medicine OR Chinese herb OR Chinese herbal medicine)

(2) CNKI (from 1979 to March 2012) (the strategies are translated from Chinese)

Fulltext：menopausal syndrome AND (Chinese medicine OR Chinese herb OR Chinese herbal medicine) AND clinical AND randomized

Title/key word/ abstract: menopausal syndrome AND (Chinese medicine OR Chinese herb OR Chinese herbal medicine)

(3) CBM (from 1978 to March 2012) (the strategies are translated from Chinese)

Key word search：

menopausal syndrome/Chinese herb OR menopausal syndrome/Chinese herbal medicine OR menopausal syndrome/integrated chinese medicine and western medicine OR menopausal syndrome/Chinese medicine

free word search：

Title/key word/ abstract: menopausal AND (Chinese medicine OR Chinese herb OR Chinese herbal medicine)

(4) Cochrane Library (from 1993 to March 2012)

(Menopaus$ OR climacteric.tw.) AND (Chinese medic$. OR Chinese herb$ OR TCM OR CHM)

(5) MEDLINE (from 1966 to March 2012) ^48^

1 exp menopause/ or exp perimenopause/ or exp postmenopause/

2 postmenopaus＄.tw.

3 perimenopaus$.tw.

4 postmenopaus$.tw.

5 menopaus$.tw.

6 exp Climacteric/

7 exp Hot Flashes/

8 (hot flush$ or hot flash$).tw.

9 climacteric.tw.

10 (vagina$ adj3 atroph$).tw.

11 (vagina$ adj3 dry$).tw.

12 night sweat$.tw.

13 sleep hyperhydrosis.tw.

14 vasomotor.tw.

15 or/1-14

16 exp Drugs, Chinese Herbal/

17 exp medicine, chinese traditional/ or exp medicine, kampo/

18 Chinese herb$.tw.

19 Chinese medic$.tw.

20 (TCM or CHM).tw.

21 herbal remed$.tw.

22 traditional chinese.tw.

23 herb$ medic$.tw.

24 or/16-23

25 15 and 24

26 randomized controlled trial.pt.

27 controlled clinical trial.pt.

28 randomized.ab.

29 placebo.tw.

30 clinical trials as topic.sh.

31 randomly.ab.

32 trial.ti.

33 (crossover or cross-over or cross over).tw.

34 or/26-33

35 (animals not (humans and animals)).sh.

36 34 not 35

37 25 and 36

2. Abbreviation list of search strategies

*ab.*, Abstract; *adj.*, adjacent, to combine different concepts in the same document; *exp.,* including all the narrower terms; *pt.*, publication type; *sh.*, subject headings; *ti.*, title; *tw.*, searched in the title and abstract fields together; *$.*, truncati
